# The Online Art Museum

**DOI:** 10.15694/mep.2020.000179.1

**Published:** 2020-08-26

**Authors:** Margot Kelly-Hedrick, Kaitlin Stouffer, Heather J Kagan, Philip Yenawine, Elizabeth Benskin, Suzy Wolffe, Margaret Chisolm

**Affiliations:** 1 Johns Hopkins University School of Medicine; 2 Watershed Collaborative; 3 The Baltimore Museum of Art

**Keywords:** arts-based teaching, visual arts, medical humanities, e-learning, coronavirus-19, art museum, COVID-19, online learning, digital, visual thinking strategies

## Abstract

This article was migrated. The article was marked as recommended.

With the onset of the coronavirus-19 (COVID-19) pandemic, we transformed an in-person art museum-based course for medical students into an online format. This brought new challenges but offered unexpected advantages. The course included daily close-looking of artworks using the Visual Thinking Strategies method, group arts-based activities, reflective writing, and independent creating assignments. The virtual format allowed us to incorporate important features that were unavailable in our in-person elective: multi-media activities, access to nearly unlimited international works of art, and personal reflection from one’s private space. As instructors, the experience enlightened us on the value of online arts-based teaching.

## The Online Art Museum

Like medical educators across the globe, we were forced quickly to adapt our educational delivery because of the coronavirus-19 (COVID-19) pandemic. We transformed an art museum-based course for medical students to an online format, which brought new challenges but offered unexpected advantages.

Our course, part of a wave of arts-based teaching in medical education ( Association of American Medical Colleges, 2020; Fancourt and Finn, 2019; Haidet *et al.,* 2016), was designed as a month-long in-person elective at The Baltimore Museum of Art (BMA) for 4 ^th^ year medical students with the goals of exploring what it means to be a physician, to be human, and to lead a good life. In March 2020, our university ceased in-person classes amidst the COVID-19 pandemic and called upon faculty to rapidly develop online electives for medical students. This prompted our team, comprised of medical school faculty (MSC), staff (MKH), trainees (HK, KS), and art museum educators (EB, SW, PY) to adapt and condense our in-person elective into a five-day course, “The Online Art Museum: Exploring Professional Identity through Art.” We ran this full-time course twice, in April and May 2020, on the Zoom videoconferencing platform, with 10 and 8 students, respectively.

Our course required two hours of in-class time on Zoom (see Table 1 for typical schedule) with additional daily out-of-class assignments. Each day’s in-class session began with a facilitated group discussion using the Visual Thinking Strategies (VTS) method, a pedagogical approach that involves a facilitated group discussion about a work of art that encourages close looking and holding space for multiple interpretations ( Yenawine, 2013). After VTS, students shared reflections on their out-of-class assignments, which included reading and creative work. We then launched into a group activity, unique each day, adapted from previously piloted museum-based activities we had run throughout the year, many designed by educators at the BMA. For example, one group activity asked students to look at a piece of art together and write a group poem in response. We closed each day’s in-class session with a reflective writing exercise and five-minute meditative exercise.

**Table 1.** Typical daily schedule for course.

*Activity*	*Length*
Visual Thinking Strategies discussion	40 minutes
“Show and tell” from previous day’s independent creating activity and discussion of assignments	30 minutes
Group activity (varies each day)	30 minutes
Reflective writing exercise	15 minutes
Meditative exercise	5 minutes

The activities in our online course were more varied than we could have accommodated in an in-person offering. We incorporated multimedia experiences of music and video in class. Some homework assignments involved finding or making objects, which students could then show to their peers over Zoom. The school-wide schedule restructuring broadened our participant base to include students across multiple class years, enhancing the diversity of student perspectives represented. The online format also expanded the content we were able to access-we selected diverse works from online museum collections around the globe and were not limited to art currently exhibited at the BMA.

Our group quickly formed what was described by one student as “a close knit and safe community.” In the virtual setting, students shared deeply personal stories and reflections. One student added that being able to participate from the security and comfort of home fostered a feeling of ease to reflect and share, leading to deepened introspection ( Table 2). In the online course, all of the students were able to have their cameras on, with the option to turn off their cameras during individual tasks such as writing exercises. This allowed individual introspection and privacy on a different level than our in-person course could offer in the public museum space.

**Table 2.** Students’ feedback about the strengths and limitations of online course format. ^*^

*Strengths*
“I thought this was the next best thing to an in-person course. I felt engaged and I appreciated being able to see and hear my colleagues and educators.”
“I am grateful we have tools and [are] able to adapt to still get to have meaningful conversations and activities. The online activities were meaningful... very powerful and inspiring.”
“I loved having the opportunity to take this course online and having everyone with their camera on in such a small group was almost like doing it in person.”
“A great way to engage from afar, the comfort of home can also facilitate reflection. Although there are benefits to being in a museum, being at home comes with unique advantages for self-reflection”
“Thought the sessions went well over Zoom. Way less awkwardness than I was expecting based on previous experiences.”
***Limitations***
“Easier to accidentally disengage [in online setting], for example to get an email and respond to it during the course as opposed to ignoring it, or not even knowing it came because you are not on your electronic device.”
“Weaknesses- not being able to meet in person, maybe a little more [of a] barrier to speak and share over video”
“I wonder if some of the images were blurry for people, based on their internet connection. During one session, the photo appeared blurry for me. It may help to check in with students to confirm that they can see the image clearly.”

* At the end of the course, we invited students to participate in an optional, online feedback survey as part of an IRB-exempted research study. Eleven of the eighteen students filled out the survey (response rate = 61%). These are some of the responses to the item: “If you have any comments about the online format of the course (strengths and/or weaknesses), please share them here.”

Students provided valuable feedback on a post-class survey ( Table 2). All respondents (n=11) expressed a desire for a formal arts-based program in their medical education. When queried about preferred format, over half (n=7/11) said they would *prefer* a mix of online and in-person activities, while two students expressed interest in an entirely online course.

Transforming art museum-based teaching to a virtual setting was a challenge-how would we recreate the magic of the art museum online? But with the help of technology, many activities worked just as well, if not better, online. The experience opened our eyes to the potential for online arts-based teaching to bring us closer while staying physically distant. On the last day of the course, students lingered past the official end time, reluctant to leave the sanctuary we had created together.

## Take Home Messages

• Art museum-based activities for medical education can succeed in an online format.
• Online arts-based teaching enables inclusion of multi-media activities, access to nearly unlimited international works of art, and private opportunities for student reflection, features not possible in a public museum setting.

## Notes On Contributors

Margot Kelly-Hedrick is a research program coordinator in the Department of Psychiatry and Behavioral Sciences at Johns Hopkins University School of Medicine. ORCID: https://orcid.org/0000-0002-2188-1244

Kaitlin Stouffer is an MD-PhD student at Johns Hopkins University School of Medicine.

Heather J Kagan is a resident in the Department of Medicine at Johns Hopkins University School of Medicine.

Philip Yenawine is the creative director at Watershed Collaborative.

Elizabeth Benskin is a museum educator in the Department of Education at The Baltimore Museum of Art.

Suzy Wolffe is a museum educator in the Department of Education at The Baltimore Museum of Art.

Margaret S. Chisolm is a professor in Department of Psychiatry and Behavioral Sciences, and of Medicine, at Johns Hopkins University School of Medicine.
