# Supplementary material for: The Online Art Museum
Source: MedEdPublish (2016). 2021 Sep 3;9:179. Originally published 2020 Aug 26. [Version 2] doi: 10.15694/mep.2020.000179.2 (PMC10702638; doi:10.15694/mep.2020.000179.2)
Supplement: Peer review history

# Reviewer report: Reviewer response for version 2

**DOI:** 10.21956/mep.20276.r31477

**Published:** 2021-09-07

**Authors:** P Ravi Shankar

**Affiliations:** 1 American International Medical University

This review has been migrated. The reviewer awarded 4 stars out of 5 Thank you for an interesting article and for the revisions. We recently used VTS during an online workshop for medical teachers in India. We used paintings by our students at the International Medical University, Malaysia. The online mode of delivery has both advantages and limitations as you have pointed out. As the pandemic persists educators are gaining a better understanding of online teaching-learning methods.

Reviewer Expertise:

NA

No decision status is available

# Reviewer report: Reviewer response for version 2

**DOI:** 10.21956/mep.20276.r31476

**Published:** 2021-09-04

**Authors:** Megan Anakin

**Affiliations:** 1 University of Otago

This review has been migrated. The reviewer awarded 4 stars out of 5 Dear Margot and colleagues, Thank you for publishing this revised version of your article. Thank you for the additional description of the programme in the introduction of this revised article. The description of students’ evaluation responses also adds more information for the reader about how the programme was appreciated by participating students. The take home messages are now well supported by the main arguments and data presented in the article. Thank you. Others who are interest in the area of medical humanities will find this article thought-provoking. I hope to see a future article from this group of authors. I would be keen to read more about how this course contributes to the development of future doctors into your medical programme and how it relates to our growing understanding about the importance of the humanities in medical education.

Reviewer Expertise:

NA

No decision status is available

# Reviewer report: Reviewer response for version 1

**DOI:** 10.21956/mep.19008.r27274

**Published:** 2020-09-05

**Authors:** Megan Anakin

**Affiliations:** 1 University of Otago

This review has been migrated. The reviewer awarded 2 stars out of 5 I read your new education method or tool article with interest because I am supervising a master’s student conducting a study about medical humanities in India. The medical programme at my university has also been impacted by changes in teaching and learning practices due to the COVID19 pandemic, therefore, I was eager to learn about how your team adapted your course to an online format. Overall, I felt that this article has the potential to be useful, however, a description of the month-long in-person elective is needed to understand the comparative statements made about how well or better the online course was appreciated by students when the authors interpreted their evaluation feedback. For example, there is no evidence provided to support the statement, “The activities in our online course were more varied than we could have accommodated in an in-person incorporated multimedia experiences of music and video in class.” and the other comparative statements in the fourth paragraph of the article, and the statement in the eighth paragraph (i.e., “But with the help of technology, many activities worked just as well, if not better, online”).To improve the usefulness of the methods described in this new education method or tool article, the authors may wish to provide examples of the reading and creative work students completed in their out-of-class assignments and the prompts used for the reflective writing exercise and five minute meditative exercise used at the closure of each day’s in-class sessions.Please describe how the evaluation feedback was analysed so the reader understand how the quotations shown in Table 2 were chosen. This explanation will help the reader to understand how the student feedback was valued in the first sentence of the seventh paragraph of the article. Please consider explaining how these evaluation findings might be used to enhance the online (or the in-person) course in the future.Please consider revising the take home messages so that they accurately reflect the evidence presented in this new education method or tool article. In the first message, please consider defining success earlier in the article and explain how success was measured or addressed by the students’ evaluation feedback. In the second message, please consider revising the absolute statement about it not being possible to include multi-media activities, access to nearly unlimited international works of art, and private opportunities for student reflection in a public museum setting because the features of the month-long in-person elective were not described to the reader nor was other evidence from the literature presented to support this claim.I would be very happy to read and review a revised version of this article.

Reviewer Expertise:

NA

No decision status is available

# Reviewer report: Reviewer response for version 1

**DOI:** 10.21956/mep.19008.r27273

**Published:** 2020-09-03

**Authors:** P Ravi Shankar

**Affiliations:** 1 American International Medical University

This review has been migrated. The reviewer awarded 4 stars out of 5 This is an interesting description of an online arts museum course for undergraduate medical students. Online teaching-learning has become important during the ongoing pandemic. AMEE MedEdPublish has published a large collection of articles on the pandemic. The authors’ initiatives are important. I would like to request the authors to provide more description about the module, the learning objectives, the actual painting used and how they were selected. A description of the out-of-class assignments would also be of interest. Online courses like these will be playing an important role in medical and health professions education for some time to come. The article will be of interest to all health humanities educators.

Reviewer Expertise:

NA

No decision status is available

# Reviewer report: Reviewer response for version 1

**DOI:** 10.21956/mep.19008.r27272

**Published:** 2020-08-30

**Authors:** Flora Smyth Zahra

**Affiliations:** 1 Faculty of Dentistry, Oral &Craniofacial Sciences. King's College London

This review has been migrated. The reviewer awarded 5 stars out of 5 As clinical educators have had to quickly learn to adapt their curricula during the pandemic, I congratulate the authors for being brave enough to transform what would have been an in-person art-museum course for medical students to the online format. For those of us who currently are pioneering health professions' educators in these cultural places and open spaces, we know the value of being in the sanctity of the gallery and museum and many might be tempted to think that much of the very essence of the experience would be lost in the transition. This succinct and helpful account will encourage others to be brave, to take the plunge and innovate in the current circumstances whether teaching in this realm or in other areas of medical education. What the authors detail from the students themselves, is that there was great benefit and much to be gained, often unexpectedly from this online shift. At present, arguably more than ever before, educators need to reach out to their students, be present and offer them time and space to think, reflect and make meaning and the art-museum in this virtual form offered the students exactly that, as it always does, supporting the students personal development as they start to acquire their professional identities. This form of clinical teaching is not easy and requires skilful curation, sensitive presentation and good collaboration between museum educators and their clinical counterparts, but this expert team have shown that in the right hands the art-museum builds relational and self-care 'power skills' that are becoming even more vital for this next generation of doctors.

Reviewer Expertise:

NA

No decision status is available
